# Relationship Between Well-Being and Inclusive Practice in Chilean Teachers: A Preliminary Analysis

**DOI:** 10.3390/jintelligence13120152

**Published:** 2025-11-22

**Authors:** Marco Villalta-Paucar, Jéssica Rebolledo-Etchepare, Juan Pablo Hernández-Ramos

**Affiliations:** 1 Escuela de Psicología, Universidad de Santiago de Chile, Santiago 9170022, Chile; jessica.rebolledo@usach.cl; 2 Instituto de Ciencias de la Educación, Universidad de Salamanca, 37008 Salamanca, Spain; juanpablo@usal.es

**Keywords:** well-being, professional development, assessment, inclusive education, life satisfaction, primary school teacher, teacher education, optimism

## Abstract

Although numerous studies address inclusive education, especially in Latin America, research analyzing the overall life satisfaction of teachers in schools that implement inclusion policies are scarce. The purpose of this study is to analyze the relationship between Life Satisfaction, Optimism, Culture, and the Inclusive Practice of primary school teachers from Chile. A descriptive quantitative method was employed, with an ex post facto design including 246 primary teachers from urban and rural schools in Chile. The teachers completed four questionnaires: Inclusive Culture (IC), Inclusive Practice (IP) Satisfaction with Life Scale (SWSL), and Life Orientation Test Revised (LOT-R). The results show that these instruments present acceptable reliability. In addition, a significant correlation was found between Classroom Experience Time (CET) and SWSL (r = 0.201, *p* < .01), as well as between SWSL, and LOT-R (r = 0.411, *p* < .01), and IC and IP (r = 0.838, *p* < .01). The regression model is statistically significant [F (4, 241) = 139.572, *p* < .001]. The findings indicate that IC and SWSL predict IP directly, whereas CET is an inverse predictor. There is a statistically significant relationship between Life Satisfaction, Classroom Experience Time, Culture, and Inclusive Practice, with the three first variables being predictors of Inclusive Practice.

## 1. Introduction

Well-being and inclusive education are both social demands and goals of contemporary social and education policies (Organización de las Naciones Unidas para la Educación la Ciencia y la Cultura [UNESCO] 2020; Ozturk et al. 2024). A review of the Web of Science (WoS) database about published scientific research on these topics reveals that, independently, the number of studies about well-being and inclusive education have sustainedly increased by thousands since the late 20th century. However, research analyzing the relationship between these two constructs is rather recent and comparatively scarce. Therefore, there is a knowledge gap that this study aims to fill.

UNESCO member countries have established agreements and legislative commitments to promote inclusive education in schools (2020). Diverse approaches address the concept of inclusive education (Abed and Shackelford 2023; Görel et al. 2024; Peña et al. 2022; Pozo-Armentia et al. 2020), and the challenge of achieving full inclusion in educational institutions (Hornby and Kauffman 2024). UNESCO’s definition serves as a guide because it conceptualizes inclusive education as a systematic change process promoted by schools to eliminate the attendance, participation, and learning barriers of all students (Organización de las Naciones Unidas para la Educación la Ciencia y la Cultura [UNESCO] 2017). This definition is operationalized through the Inclusion Index proposed by Booth and Ainscow (Ainscow et al. 2006; Booth and Ainscow 2000) and has been validated in different studies and countries (Jashes Morgues et al. 2024; Losada-Puente et al. 2023; Fiuza-Asorey et al. 2021; Campa-Álvarez et al. 2020; Castillo Armijo et al. 2020).

Most studies on inclusive education in primary education have focused on students with special education needs (Abed and Shackelford 2023; Bendová and Fialová 2015; Hornby and Kauffman 2024), as well as on the attitudes of teachers towards the inclusion of these students (Radojlovic et al. 2022; Schmidt et al. 2024; Stemberger and Kiswarday 2018). Research has also addressed the perceptions about the inclusion of migrant and refugee children (Besic et al. 2020), students on the autism spectrum (Raudeliunaite and Steponeniene 2020; Šilc et al. 2024), cultural diversity (Bustos-Rubilar et al. 2021; Martínez-Santa et al. 2025; Muhammad and Liu 2025), and linguistic diversity and migrants (Liakou 2023; Morin et al. 2025). Recently, research has also focused on students from sexual minorities, considering their impact on learning and well-being (Arias-Pastor et al. 2024; Heyder et al. 2020).

School Culture refers to the daily management and behavior of the members of the educational community, students, teachers, leadership, and management staff at school and in the classroom. It is shaped by beliefs and shared routines that are confirmed and changed through reflection on the practice (Martinic Valencia et al. 2024; Villalta et al. 2023). In this sense, Inclusive Culture is a paradigm shift in school relationships, referring to positive approaches towards classroom diversity, participation, and learning of all students (Booth and Ainscow 2000; Juan-Morera et al. 2023; Valle-Florez et al. 2022); this variable experiences the tension between attention to diversity of students in the classroom or the integration that discriminate students who are different (Valle-Florez et al. 2022). In turn, Inclusive Practice is the most visible and palpable elements of inclusion, given that practices make policies and culture concrete (Juan-Morera et al. 2023); nevertheless, the relationship between Culture and Inclusive Practice is not automatic and is mediated by teacher reflection and self-efficacy (Villalta Paucar et al. 2025; Wang et al. 2022; Woodcock et al. 2022), as well as by having adequate resources to address diversity (Juan-Morera et al. 2023). In light of the above, promoting an Inclusive Culture at schools implies fostering shared values, reflecting on practice, teacher self-efficacy, and necessary resources to cater to student diversity.

The achievement of inclusive education in schools is enabled by the positive attitudes and emotions of teachers (Görel et al. 2024; Huang 2023; López et al. 2010), their reflection on the teaching practice, and the resources available that influence teacher self-efficacy perception (Arias-Pastor et al. 2024; Görel et al. 2024; Stemberger and Kiswarday 2018; Goldan et al. 2021; Villalta-Paucar et al. 2025), as well as the collaborative work with other professionals and the community (Kurniawati 2021; Petri et al. 2025; Wiedebusch et al. 2022); all of this must be performed without neglecting either the initial or continued training of teachers (Hernandez-Ramos and Martinez-Abad 2023). In fact, the values and beliefs of teachers are key aspects to achieve educational and pedagogical inclusion in schools and classrooms (Florian and Beaton 2018; Huang 2023; Orozco and Moriña 2023). It can be assumed that the success of inclusive education is related to the educational communities—students, teachers and professionals—that maintain adequate levels of well-being.

Teachers who work with students with special educational needs present levels of depersonalization that affect their occupational well-being and lead to emotional exhaustion, one of the main symptoms of the burnout syndrome (Elsayed et al. 2025; Saloviita and Pakarinen 2021). Teacher self-efficacy favors educational inclusion and the perception of self-fulfillment, and increases when teachers interact with people with disabilities (Vieira et al. 2024; Wray et al. 2022) and when there is distributed and transformational leadership by management and teaching leadership in the inclusive classroom (Wang et al. 2024; Wang et al. 2022). Adequate diversity management at school fosters a good organizational climate, which guarantees teachers’ high work satisfaction and positive emotions about their work (Otrebski 2022), but does not significantly moderate the interaction between attention to students with special educational needs and the emotional exhaustion of teachers in inclusive classrooms; furthermore, studies indicate that work commitment, work overload, and burnout occur simultaneously in inclusive classrooms (Kuok et al. 2020; Weißenfels et al. 2021). In fact, research has found that, alongside teacher self-efficacy, other influential institutional and contextual factors (Elsayed et al. 2025), such as coworker and management leadership, personality traits, and personal life events and changes—elements that suggest that future studies on burnout should consider its multifaceted nature (Elsayed et al. 2025; Saloviita and Pakarinen 2021). Thus, a comprehensive understanding of the burnout phenomenon requires an examination of how Inclusive Practice in schools is related to Inclusive Culture, and also a broader perspective on well-being, as virtuous cycles that foster effective inclusion influences the mental health of teachers.

In turn, well-being has been researched from diverse theoretical and empirical perspectives, focusing on specific groups. In teacher well-being studies, the concept is defined as the predominance of positive appraisals of the teaching work, the consequence of personal traits, and the interaction context in the educational organization (Dávila et al. 2024; Reppa et al. 2023). A systematic review of the scientific literature indicates that the study of teacher well-being focuses on the occupational dimension in relation to self-efficacy and resilience (Arias-Pastor et al. 2024; Gilar-Corbi et al. 2025; Reppa et al. 2023), overlooking a comprehensive approach that considers all aspects of well-being, including the teaching profession itself (Ozturk et al. 2024). From this perspective, overall Life Satisfaction allows for people to assess all of the dimensions and stages of life in their affective and cognitive terms, according to personal expectations (Diener et al. 1985; Espejo et al. 2022). This construct is measured with the Satisfaction with Life Scale (SWSL) designed by Diener et al. (1985), widely used in psychological research, which has demonstrated adequate reliability in diverse cultural contexts, including the Spanish-speaking community (Bahamón et al. 2025; Jurado et al. 2019; Liu et al. 2025; Vera-Villarroel et al. 2012). In addition, a relationship has been determined between the overall Life Satisfaction and subjective well-being of teachers (Muñoz-Campos et al. 2018).

The beliefs and positive attitudes towards inclusive education enable the actions necessary to achieve it (Huang 2023; Orozco and Moriña 2023). From a positive psychology perspective, this can be explained through the dispositional optimism construct (Carver and Scheier 2001), which is understood as the generalized expectations for the occurrence probability of favorable or positive events (Ortiz et al. 2016; Rondón Bernard and Angelucci Bastidas 2016). This construct has been measured using the Life Orientation Test-Revised or LOT-R (Scheier et al. 1994), which explains the attitude of people towards the future in all the dimensions of their life. LOT-R is an instrument widely used in psychological research to assess Optimism (Ortiz et al. 2016; Rondón Bernard and Angelucci Bastidas 2016; Vera-Villarroel et al. 2009). A systematic review indicates that higher levels of Optimism may bring benefits for physical and mental health, and LOT-R acts as an important moderator of positive expectations (Malouff and Schutte 2017).

Teachers lead the educational process and, consequently, are indispensable in the inclusive education process. Life Satisfaction and the optimistic disposition of teachers must be key aspects to facilitate the transition from homogeneity-based teaching to inclusive management that acknowledges and addresses heterogeneity in the classroom. However, the social changes experienced in recent decades have negatively impacted teacher well-being, reducing the acknowledgement of their role and generating sometimes unfair work conditions, including work overload and challenges for developing socioemotional skills that enable them to take care of the diverse needs of their students (Arbia et al. 2023). Teaching is among the professions with the highest risk for mental health issues due to work overload (Ródio Trevisan et al. 2022), the perception of lacking the tools for addressing the specific educational needs of students (Schmidt et al. 2024), the social devaluation of the teaching work, inadequate conflict management, and the precarity or absence of resources. Therefore, it can be implied that, in contexts where teacher malaise is predominant, inclusive education is unfeasible.

Life satisfaction reflects and permeates the emotions associated with daily actions in the diverse aspects of teaching practice. However, no studies were found that address inclusive education and teacher Life Satisfaction. This study aims to analyze the relationship between inclusive education and the Life Satisfaction, Optimism, Culture, and Inclusive Teaching Practices of primary school teachers from Chile. The main conclusions are as follows: (1) a positive and significant correlation was found between Life Satisfaction, Optimism, Inclusive Culture, and Inclusive Practice scales; (2) the length of Classroom Experience Time is directly correlated with Life Satisfaction and inversely correlated with Inclusive Practice; and (3) Life Satisfaction, Classroom Experience Time, and School Culture are the variables that predict Inclusive Practice.

## 2. Materials and Methods

### 2.1. Participants

The participants were teacher and management teams from 12 primary schools located in urban and rural sectors of three Chilean regions, namely Metropolitana, La Araucanía, and Aysén. The southern regions (La Araucanía and Aysén) present the rural schools that serve populations with the highest socioeconomic poverty indicators according to the School Vulnerability Index (IVE), which reports the official numbers of the Government of Chile (Junta Nacional de Auxilio Escolar y Becas [JUNAEB] 2025).

Sampling was conducted using a simple non-probabilistic randomized procedure. The sample was composed of 246 participants whose ages ranged from 23 to 69 years (mean = 42 years). Teaching experience varied from 1 to 44 years (mean = 14 years). Of the participants, 187 were women (54 in rural schools and 133 in urban schools) and 59 were men (15 in rural and 44 in urban schools, respectively). Regarding their roles at school, 189 participants were classroom teachers—142 women, 47 men—30 worked as education support professionals—25 women, 5 men—and 27 were part of the management team—20 women and 7 men. All were generalist teachers, i.e., they taught all of the subjects to the same group of students. In conclusion, the sample was diverse in age and professional experience, with a predominance of women in the direct classroom teaching roles at the primary level of the participating schools.

### 2.2. Instruments

#### 2.2.1. Inclusive Culture Questionnaire (IC) by Booth and Ainscow (2000) with 12 Likert-Type Items Ranging from 1 (Totally Disagree) to 4 (Totally Agree)

This instrument assesses appraisals about the achievements of the educational community based on shared values and beliefs about inclusive education, with items such as “The school works with organizations from the community” [our own translation]. Studies have already assessed the reliability of this index in Chilean population (Jashes Morgues et al. 2024).

#### 2.2.2. Inclusive Practice Questionnaire (IP), Booth and Ainscow (2000), with 17 Likert-Type Items Ranging from 1 (Totally Disagree) to 4 (Totally Agree)

This instrument measures whether classroom activities promote the participation of all students and overcome the existing barriers through items such as “Teachers are concerned with supporting the learning and participation of all students” [our own translation]. The reliability of this scale has been studied in Chilean population (Jashes Morgues et al. 2024).

#### 2.2.3. Satisfaction with Life Scale (SWLS) by Diener et al. (1985) with 5 Likert-Type Items Ranging from 1 (Totally Disagree) to 7 (Totally Agree)

This scale assesses overall life satisfaction with the own life. It has a version adapted to the Chilean population, which has demonstrated a Cronbach’s alpha internal consistency = 0.82 (Vera-Villarroel et al. 2012).

#### 2.2.4. Life Orientation Test, Revised LOT-R, Version by Scheier et al. (1994), Including 10 Items: 6 Assessing the Content and 4 Assessing Distractors

It measures the degree of Optimism or Pessimism of individuals in a 5-level Likert scale, where 0 indicates “strongly disagree” and 4 “strongly agree”. Of the 6 main items, 3 are written in positive direction, while the remaining 3 are in the negative direction for Pessimism. To calculate the total score, the negatively worded items are reverse-scored so that a higher score reflects greater Optimism. LOT-R has been adapted and validated in the Chilean population, obtaining a Cronbach’s alpha = 0.65 (Vera-Villarroel et al. 2009).

### 2.3. Procedure

The data collection procedure was conducted online between October 2024 and September 2025. Ethical protocols validated by a Research Ethics Committee accredited in the country were followed. Schools were informed, and their permission was first obtained to then contact them in person and via e-mail. An informed consent letter was presented to them that guaranteed voluntary participation and response confidentiality. The estimated duration to complete the instruments was 15 min.

The data analysis was conducted by calculating the main descriptive statistics (mean and standard deviation) in order to achieve a comprehensive sample characterization and to synthesize the central properties of the construct variables (Inclusive Culture, Inclusive Practice, Satisfaction with Life, and Optimism). Likewise, to ensure the psychometric robustness of the instruments, the reliability of the four measurement scales was assessed using Cronbach’s alpha, a statistical technique that guarantees the internal consistency and consistency with which items measure the latent construct and an indispensable methodological requirement for any subsequent analysis. Given that the study variables (Classroom Experience Time, Inclusive Culture, Inclusive Practice, Satisfaction with Life, Optimism, and Pessimism) are conceptualized and operationalized as continuous variables, Pearson’s correlation coefficient (r) was used to examine the bivariate associations among them. To conclude, Levene’s test was used to verify the homogeneity of the variance, and then a multiple linear regression analysis was conducted to determine the predictive capacity of the independent variables (Classroom Time Experience, Inclusive Culture, Satisfaction with Life, and LOT-R) over the dependent variable Inclusive Practice.

## 3. Results

The reliability of the instruments varies: the Inclusive Practice Questionnaire presents an excellent or very good reliability, with a Cronbach’s alpha of 0.932; the Inclusive Culture Questionnaire has good reliability, with a Cronbach’s alpha of 0.888; and the Satisfaction with Life Scale also exhibits good reliability, with a Cronbach’s alpha of 0.868. However, LOT-R has poor reliability, with a Cronbach’s alpha of 0.550. Nevertheless, when analyzing the Optimism direction, the reliability is acceptable (Cronbach’s alpha: 0.727), while in the Pessimism-direction items, the reliability is not acceptable (Cronbach’s alpha: 0.424) (see Table 1). These results, which are also confirmed by other studies using the LOT-R scale, suggest the importance of differentiating between the Optimism and Pessimism dimensions (Rondón Bernard and Angelucci Bastidas 2016; Vera-Villarroel et al. 2009).

Pearson’s correlation between Inclusive Culture and Inclusive Practice instruments is strong, positive, and statistically significant (r = 0.838, *p* < .01), confirming the interdependence between both in the context of inclusive education. Classroom Experience Time (CET) is significantly and positively correlated with Life Satisfaction (r = 0.201, *p* < .01) and has a negative and statistically significant relationship with Inclusive Practice (r = −0.147, *p* < .05). In turn, despite the weak correlation of Inclusive Practice with Life Satisfaction, Life Satisfaction shows a positive and significant relationship with Inclusive Culture (r = 0.212, *p* < .01) and with Inclusive Practice (r = 0.213, *p* < 0.01), as well as a moderate correlation with LOT-R (r = 0.411, *p* < .01). Optimism presents a weak, positive, and significant correlation with Inclusive Culture (r = 0.240, *p* < .001) and Inclusive Practice (r = 0.236, *p* < .01) and a moderate, positive, and significant correlation with Satisfaction with Life Scale (r = 0.491, *p* < .01). Meanwhile, Pessimism exhibits no correlation with Classroom Experience Time, Optimism, and Satisfaction with Life Scale, but presents negative and significant correlations with Inclusive Culture (r = −0.200, *p* < .01) and Inclusive Practice (r = −0.217, *p* < .01). These results support the theoretical constructs addressed by the instruments used in this study (see Table 1).

A multiple linear regression analysis was conducted in which the predictor variables were ET, IC, SWSL, and LOT-R, while the predicted variable was Inclusive Practice. Levene’s test indicated heterogeneity of variances between the men and women groups [F(1, 244) = 2.149, *p* = .023]; therefore, the linear regression was conducted by weighing the variable sex. The regression model is statistically significant [F (4, 241) = 139.572, *p* < .001] with a R^2^ = 0.698, which indicated that approximately 70% of the variance in Inclusive Practice can be explained in its relationship with the predictor variables. The variables that resulted statistically significant as predictors were Classroom Experience (B = −0.062, EE = 0.030, *p* = 0.040), Inclusive Culture (B = 1.233, EE = 0.056, *p* < .001), and Life Satisfaction (B = 0.151, EE = 0.076, *p* = .048). The other predictor variables did not show statistical significance (*p* > .05); see Table 2 and Table 3.

Table 2 shows the descriptions of the variables considered in the regression analysis.

The relationship observed between Classroom Experience and Inclusive Practice is of negative direction. This confirms the findings reported in Table 1: the more the Classroom Experience, the lower the perception of Inclusive Teaching Practices, while the less Classroom Experience, the more the sensitivity to observe such practices. In addition, both Life Satisfaction and Inclusive Culture predict directly and positively the score of the Inclusive Practice Questionnaire, i.e., the perception and self-perception of Inclusive Practices.

## 4. Discussion

This study contributes to our understanding of the relationship between Life Satisfaction, Optimism, Culture, and Inclusive Practice in teachers from primary schools of Chile, based on evidence collected with reliable instruments. The questionnaires about Inclusive Culture and Practice are part of the Inclusion Index by Booth and Ainscow (2000) and support the self-assessment process of schools in their path to inclusive education. Furthermore, new empirical evidence is presented, which supports the good and excellent reliability of both questionnaires for the Chilean population, confirming previous results from Mexico (Campa-Álvarez et al. 2020) and Chile (Castillo Armijo et al. 2020). In turn, the Satisfaction with Life Scale by Diener et al. (1985) shows good reliability between the teachers, management, and educational support professionals participating in the study. This finding updates and confirms the results of previous studies about the reliability of this scale in different populations, such as Chilean university students (Vera-Villarroel et al. 2012), children, adolescents, and a Colombian population (Bahamón et al. 2025), and Mexican university students (Jurado et al. 2019).

Some elements should be considered when using the LOT-R. The six-item test applied to primary school teachers showed low statistical reliability, lower than that reported in similar studies conducted in Chile with university students (Ortiz et al. 2016; Vera-Villarroel et al. 2009) and similar to that found in a Venezuelan adult population (Rondón Bernard and Angelucci Bastidas 2016). Some studies suggest that the LOT-R can be addressed in two independent factors, namely Optimism and Pessimism (Rondón Bernard and Angelucci Bastidas 2016; Vera-Villarroel et al. 2009), being recommended to measure these two constructs separately (Malouff and Schutte 2017). From this perspective, this study contributes evidence on the bidimensional character of the LOT-R; in this teacher sample, the Optimism subscale showed acceptable reliability, whereas the Pessimism subtest did not.

Consequently, the data of this study indicates good and acceptable reliability in all of the instruments employed, which agrees with previous studies with other samples and countries. This allows us to address these analyses and results with more certainty. In this sense, the correlation analyses reveal that all of the analyzed variables confirmed the theoretical expectations. Specifically, this study provides evidence for a high correlation between Inclusive Culture and Practice (r = 0.838), which is consistent with the findings in a Mexican sample where r = 0.741 (Campa-Álvarez et al. 2020) and indicates a significant, strong, and bidirectional relationship between Inclusive Culture and Practice.

The Classroom Experience Time of teachers exhibits a weak, negative, but significant correlation with Inclusive Practice. This suggests that teachers with less Classroom Experience tend to show more disposition to acknowledge inclusive educational practices in their schools, while this disposition is significantly lower between those with more experience in the classroom.

SWLS presents a positive and statistically significant correlation with Inclusive Culture and Practice. This coincides with previous qualitative studies that indicated Community Support, Positive Climate, and Empathy as essential characteristics related to inclusion (Görel et al. 2024; Huang 2023), despite not specifically assessing Life Satisfaction. In this sense, this study contributes to this finding through a specific assessment of this variable and contributes to strengthening the evidence line already reported by other authors.

Likewise, the SWLS correlated moderately and significantly with the LOT-R, which is consistent with the theoretical constructs of both instruments. In fact, a positive life appraisal is related to positive expectations about the future. This study empirically evidences that overall Life Satisfaction has a significant relationship with dispositional Optimism, and both variables exhibit a significant relationship with Inclusive Culture and Practice. This finding contributes to considering well-being from a wide perspective and are not only associated with the professional role at school (Ozturk et al. 2024).

In turn, the dimension of Pessimism on the LOT-R, despite presenting very low internal reliability, shows external validity in its negative correlation with the Inclusive Culture and Practice questionnaires. In fact, the available data of this study indicated that adopting low positive expectations goes in the opposite direction of the perception of a collaborative school community and the development of Inclusive Teaching Practice.

This study also reveals specific and direct relationships between positive variables traditionally research in the health, clinical, and social sciences fields, but not specifically in the field of inclusion and Inclusive Practice of school community members. In this sense, this study shows the relationship between Life Satisfaction, Optimism, Culture, and Inclusive Practice in education.

**Figure 1:**
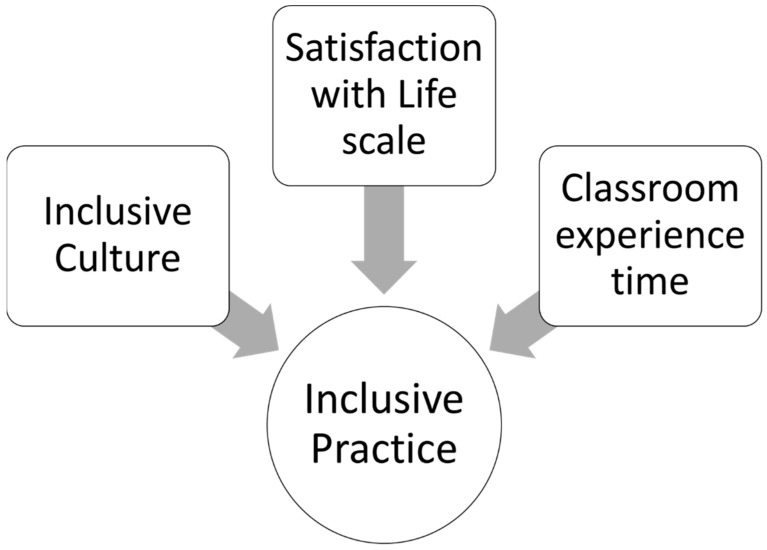
Predictor variables of Inclusive Practice in Chilean primary school teachers.

To explore possible causal and predictive relationships, a regression analysis was conducted. The results indicate that the variables of Classroom Experience Time, Life Satisfaction, and Inclusive Culture predict Inclusive Practice significantly and in different directions (Figure 1).

The available data demonstrate that Inclusive Culture and Life Satisfaction are conditions for the development of Inclusive Practice. In this sense, elements, such as reflection on the own classroom practice (Villalta Paucar et al. 2025), collaborative work with other professionals, community, and well-being associated with self-efficacy (Petri et al. 2025), contribute to the perception of Inclusive Practice in educational institutions.

In turn, Classroom Experience Time predicts Inclusive Practice in an inverse direction: the longer the time working in the classroom, the lower the disposition to perceive Inclusive Practice, whereas teachers with fewer years of experience are more receptive to observing such practices. In addition, the more experience in the classroom, the more Satisfaction with Life.

These results open up new hypotheses that should be explored in further studies, such as the possible influence of social desirability in the responses of teachers with less experience in the classroom. It is also hypothesized that longer teaching practice could become a risk for mental health (Ródio Trevisan et al. 2022), considering that changes in classroom realities have generated an overload in teaching practices (Arbia et al. 2023). Likewise, it is also possible that teachers with more experience in the classroom and overall Life Satisfaction have the conditions for making critical appraisals about Inclusive Practice without it affecting their commitment to inclusive education, with this being an explanatory hypothesis derived from the results of this study.

The practical implications of this study are significant and varied. The findings contribute to the design of educational policies that prioritize teacher well-being as a fundamental pillar for the promotion of educational inclusion at schools. In addition, this opens up the possibility to develop and implement intervention programs aimed at improving teacher well-being by offering training in coping strategies, mindfulness, and effective Inclusive Practice. These results are also invaluable for the initial (or pre-service) and continuous training of teachers, as well as raising awareness about the importance of well-being and providing tools for maintaining it in the context of inclusion. Finally, this study can contribute to the creation of more positive and supportive organizational climates at schools, promoting collaboration and the exchange of experience and leadership that adds value and fosters the well-being of all teaching staff.

This paper lays the foundation for a series of future studies. There is a growing need for more longitudinal studies that enable an understanding of the temporal dynamics between teacher well-being, Inclusive Culture, and implemented practices, analyzing how these elements influence one another over time. Enriching this panorama with qualitative methodologies would facilitate the in-depth exploration of teacher experiences, as well as the challenges intrinsic to Inclusive Practice and the strategies teachers adopt to take care of their own well-being. Comparing diverse educational contexts, such as urban and rural schools or different educational systems, could reveal the cultural and contextual factors that modulate these relationships. Lastly, the development and implementation of intervention studies focused on strengthening both teacher well-being and Inclusive Culture offer the opportunity to assess the effectiveness of support programs and strategies.

## 5. Conclusions

The results show a moderately significant relationship between overall Life Satisfaction and Optimism, both exhibiting a positive but low statistically significant correlation with Inclusive Culture and practices. Well-being comprises, but is not limited to, the school space, and is related to a positive appraisal of the cultural aspects of the school, such as maintaining high expectations (especially for students) and effort to reduce discriminatory practices. This is also linked to Inclusive Practice, such as concern with supporting the learning and the participation of all students.

This study confirms that Inclusive Culture and Life Satisfaction are key conditions for the best development of Inclusive Practice. Furthermore, Classroom Experience Time is related to overall life satisfaction and inversely predicts Inclusive Practice. These trends require further research to confirm and deepen this analysis (Figure 1).

The limitations of this study are the size of the sample and its non-probabilistic composition, which restrict the generalization of its conclusions. In addition, although the relationships found are significant, their magnitude is low, and, therefore, the results should be interpreted with caution. Nevertheless, the evidence gathered reveals relationships between the overall well-being of teachers and inclusive education that have not been studied, creating new research opportunities in other contexts. Likewise, these findings can guide the psychoeducational intervention to promote well-being from a wide perspective, considering it to be an essential component in efforts for achieving inclusive education.

## Figures and Tables

**Table 1:** Pearson’s correlation between the study instruments and Classroom Experience Time of participants (n = 246).

	CET	IC	IP	SWSL	LOT-R	Optimism	Pessimism
CET	1						
IC	−0.098	1					
IP	−0.147 *	0.838 **	1				
SWSL	0.201 **	0.212 **	0.213 **	1			
LOT-R	0.098	0.015	0.000	0.411 **	1		
Optimism	0.069	0.240 **	0.236 **	0.491 **	0.700 **	1	
Pessimism	0.073	−0.200 **	−0.217 **	0.123	0.754 **	0.059	1

Note: CET = Classroom Experience Time (in years); IC = Inclusive Culture; IP = Inclusive Practice; SWSL = Satisfaction with Life Scale; LOT-R = Life Orientation Test Revised. **. Correlation is significant at 0.01 (bilateral); *. Correlation is significant at 0.05 (bilateral).

**Table 2:** Predictive descriptive variables of the regression model.

	Minimum	Maximum	Mean	Standard Deviation
Classroom Experience Time (in years)	1	44	14.24	10.81
Inclusive Culture	19	50	37.87	6.04
Inclusive Practice	27	68	51.15	9.28
Satisfaction with Life Scale	11	35	28.72	4.78
Life Orientation Test Revised	6	24	16.94	3.44

**Table 3:** Regression analysis of the Inclusive Practice variable.

Model	B	Error Dev.	Beta	t	Sig.
(Constant)	3.134	2.765		1.134	0.258
Satisfaction with Life Scale	0.151	0.076	0.081	1.984	0.048
Inclusive Culture	1.233	0.056	0.808	22.099	0.000
Classroom Experience Time (in years)	−0.062	0.030	−0.075	−2.066	0.040
Life Orientation Test Revised	−0.121	0.102	−0.046	−1.186	0.237

## Data Availability

The data presented in this study are available on request from the corresponding author to protect the confidentiality of the participants.
